# Supplementary material for: Predictors and consequences of HIV status disclosure to adolescents living with HIV in Eastern Cape, South Africa: a prospective cohort study
Source: J Int AIDS Soc. 2022 May 11;25(5):e25910. doi: 10.1002/jia2.25910 (PMC9092159; doi:10.1002/jia2.25910)
Supplement: Supplementary file 1 — Figure S1. Conceptual framework Table S1. Characteristics of study participants stratified by awareness of their HIV‐positive status at the three study rounds Table S2. Differences in demographic variables between individuals with and without viral load results at all study timepoints. Table S3. Differential change in the odds of reporting any symptom of anxiety, depression and suicidality or ART adherence between rounds 2 (R2) and 3 (R3), between those who became aware of their HIV status between surveys, versus those remaining unaware of their status. [file JIA2-25-e25910-s001.docx]

**Predictors and consequences of HIV status disclosure to adolescents living with HIV in Eastern Cape, South Africa: a prospective cohort study – Supplementary material**

Olanrewaju Edun, Yulia Shenderovich, Siyanai Zhou, Elona Toska, Lucy Okell, Jeffrey W Eaton, Lucie Cluver

**Content**

**Figure S1:** Conceptual framework

**Table S1.** Characteristics of study participants stratified by awareness of their HIV-positive status at the three study rounds

**Table S2:** Differences in demographic variables between individuals with and without viral load results at all study timepoints.

**Table S3.** Differential change in the odds of reporting any symptom of anxiety, depression and suicidality or ART adherence between rounds 2 (R2) and 3 (R3), between those who became aware of their HIV status between surveys, versus those remaining unaware of their status.

**Figure S1: Conceptual framework**

Control variables

- Age
- Age at ART initiation (HIV-treatment related outcomes only)
- Dwelling location
- Household poverty
- Orphanhood status
- Relationship with primary caregiver
- Abuse – emotional and physical
- Stigma – anticipated and secondary

Outcome variables

Mental health problem symptoms:

- Depression
- Anxiety
- Suicidality

HIV-treatment related outcomes:

- ART adherence
- Viral load suppression

Explanatory variable

HIV status disclosure

**Table S1. Characteristics of study participants stratified by awareness of their HIV-positive status at the three study rounds**

|  | Study round | | | | | | | | |
| --- | --- | --- | --- | --- | --- | --- | --- | --- | --- |
|  | **Round 1 (N = 813)** | | | **Round 2 (N = 769)** | | | **Round 3 (N = 729)** | | |
|  | **Unaware**  **n = 300** | **Aware**  **n = 513** | **p-value** | **Unaware**  **n = 151** | **Aware**  **n = 618** | **p-value** | **Unaware**  **n = 106** | **Aware**  **n = 623** | **p-value** |
| Age in years, mean (SD) | 11.5 (1.76) | 13.6 (2.40) | <0.0001 | 12.5 (1.48) | 14.8 (2.41) | <0.0001 | 13.6 (1.56) | 15.8 (2.45) | <0.0001 |
| Age in years at ART initiation, mean (SD) | 6.5 (3.54) | 8.1 (4.16) | <0.0001 | 6.4 (3.26) | 7.7 (4.13) | <0.0001 | 6.3 (3.33) | 7.6 (4.08) | 0.0004 |
| Female, n (%) | 149 (49.7) | 261 (50.9) | 0.8 | 71 (47.0) | 319 (51.6) | 0.4 | 51 (48.1) | 318 (51.0) | 0.7 |
| Urban dwelling, n (%) | 203 (67.7) | 408 (79.5) | 0.0002 | 90 (59.6) | 491 (79.6) | <0.0001 | 59 (55.7) | 497 (79.8) | <0.0001 |
| Caregiver is biological parent, n (%) | 149 (49.7) | 199 (38.8) | 0.003 | 61 (40.4) | 235 (38.0) | 0.7 | 44 (41.5) | 233 (37.4) | 0.5 |
| Household poverty, n (%) | 195 (65.0) | 336 (65.5) | 0.9 | 128 (84.8) | 461 (74.6) | 0.01 | 68 (64.2) | 416 (66.8) | 0.7 |
| Any parental loss, n (%) | 146 (48.7) | 356 (69.4) | <0.0001 | 63 (41.7) | 422 (68.3) | <0.0001 | 61 (57.5) | 456 (73.2) | 0.0002 |
| Any emotional abuse in last year, n (%) | 65 (21.7) | 137 (26.7) | 0.1 | 25 (16.6) | 176 (28.5) | 0.004 | 16 (15.1) | 122 (19.6) | 0.3 |
| Any physical abuse in last year, n (%) | 99 (33.0) | 162 (31.6) | 0.7 | 34 (22.5) | 173 (28.0) | 0.2 | 19 (17.9) | 91 (14.6) | 0.5 |
| Any anticipated stigma reported, n (%) | 64 (21.3) | 134 (26.1) | 0.1 | 24 (15.9) | 131 (21.2) | 0.2 | 12 (11.3) | 103 (16.5) | 0.2 |
| Any secondary stigma in last year, n (%) | 45 (15.0) | 105 (20.5) | 0.06 | 9 (6.0) | 54 (8.7) | 0.3 | 1 (0.9) | 26 (4.2) | 0.2 |
| Self-reported past week ART adherence, n (%) | 197 (65.7) | 362 (70.6) | 0.2 | 107 (70.9) | 410 (66.3) | 0.3 | 88 (83.0) | 480 (77.0) | 0.2 |
| Any depression symptom in past two weeks, n (%) | 126 (42.0) | 226 (44.1) | 0.6 | 43 (28.5) | 217 (35.1) | 0.2 | 33 (31.3) | 172 (27.6) | 0.5 |
| Any anxiety symptom in past month, n (%) | 185 (61.7) | 320 (62.4) | 0.9 | 30 (19.9) | 188 (30.4) | 0.01 | 25 (23.6) | 158 (25.4) | 0.8 |
| Any suicidality symptom in past month, n (%) | 11 (3.7) | 35 (6.8) | 0.09 | 0 (0.0) | 24 (3.9) | 0.03 | 0 (0.0) | 24 (3.9) | 0.08 |

^†^ Type of dwelling data missing for 1 (0.1%) adolescent at round 2.

**Table S2: Differences in demographic variables between individuals with and without viral load results at all study timepoints.**

|  | Round 1  (N = 813)  Mean(SD) or n (%) | |  | | Round 2  (N = 769)  Mean(SD) or n (%) | | | |  | | Round 3  (N = 729)  Mean(SD) or n (%) | |  |
| --- | --- | --- | --- | --- | --- | --- | --- | --- | --- | --- | --- | --- | --- |
|  | **With result**  **(n = 408)** | **Without result**  **(n = 405)** | | **p-value** | | **With result**  **(n = 331)** | **Without result**  **(n = 438)** | **p-value** | | **With result**  **(n = 118)** | | **Without result**  **(n = 611)** | **p-value** |
| Age in years | 12.6  (2.35) | 13.1  (2.44) | | 0.008 | | 14.0  (2.27) | 14.6  (2.52) | <0.0001 | | 15.0  (2.20) | | 15.5  (2.49) | 0.03 |
| Female | 207  (50.7) | 203  (50.1) | | 0.9 | | 153  (46.2) | 237  (54.1) | 0.06 | | 63  (50.1) | | 306  (53.4) | 0.6 |
| Aware of HIV status | 249  (61.0) | 264  (65.2) | | 0.3 | | 257  (77.6) | 361  (82.4) | 0.1 | | 96  (81.4) | | 527  (86.3) | 0.2 |
| Caregiver is biological parent | 185  (45.3) | 163  (40.2) | | 0.2 | | 125  (37.8) | 171  (39.0) | 0.8 | | 45  (38.1) | | 232  (38.0) | 1 |

**Table S3. Differential change in the odds of reporting any symptom of anxiety, depression and suicidality or ART adherence between rounds 2 (R2) and 3 (R3), between those who became aware of their HIV status between surveys, versus those remaining unaware of their status.**

|  | n (%) | | | | ^†^Differential change estimate (95%CI) | |
| --- | --- | --- | --- | --- | --- | --- |
|  | **No disclosure R2-R3**  **(n = 106)** | | **Disclosure R2-R3**  **(n = 40)** | |  |  |
|  | **Round 2** | **Round 3** | **Round 2** | **Round 3** | **Crude** | ^‡^**Adjusted** |
| Anxiety | 22  (20.8) | 25  (23.6) | 7  (17.5) | 8  (20.0) | 1.00 (0.27 – 3.73) | 1.01 (0.26 – 3.99) |
| Depression | 31  (29.2) | 33  (31.1) | 7  (17.5) | 12  (30.0) | 1.85 (0.56 – 6.41) | 1.92 (0.56 – 6.98) |
| ^¶^Suicidality | 0  (0.0) | 0  (0.0) | 0  (0.0) | 1  (0.03) | - | - |
| ART adherence | 75  (70.8) | 88  (83.0) | 29  (72.5) | 33  (82.5) | 0.88 (0.25 – 3.19) | 0.87 (0.24 – 3.18) |

***p ≤ 0.001, **p ≤ 0.01, *p ≤ 0.05. ^†^The differential change estimate represents the ratio of the odds ratios of reporting any symptom of anxiety, depression and suicidality or ART adherence between R1 and R2 between those who became aware vs. those remaining unaware.

^‡^Adjusted for age, sex, dwelling location, household poverty, caregiver relationship, orphanhood status, physical and emotional abuse, and anticipated and secondary stigma.

^¶^Model for suicidality failed to converge due to zero value in cell.
